# The ion channel TRPM4 in murine experimental autoimmune encephalomyelitis and in a model of glutamate-induced neuronal degeneration

**DOI:** 10.1186/s13041-018-0385-4

**Published:** 2018-07-11

**Authors:** Beatrice Bianchi, Paul A. Smith, Hugues Abriel

**Affiliations:** 1 Institute of Biochemistry and Molecular Medicine, and Swiss National Centre of Competence in Research (NCCR) TransCure, University of Bern, Bühlstrasse 28, 3012 Bern, Switzerland; 2 Autoimmunity, Transplantation and Inflammation, Novartis Institutes for BioMedical Research, Basel, Switzerland

**Keywords:** TRPM4, Experimental autoimmune encephalomyelitis, Glutamate-induced neurodegeneration, Multiple sclerosis, Inhibitors, HT22

## Abstract

Transient receptor potential melastatin member 4 (TRPM4), a Ca^2+^-activated nonselective cation channel, has been found to mediate cell membrane depolarization in immune response, insulin secretion, cardiovascular diseases, and cancer. In murine experimental autoimmune encephalomyelitis (EAE), TRPM4 deletion and administration of glibenclamide were found to ameliorate clinical symptoms and attenuate disease progression. However, the exact role of TRPM4 in EAE, as well as the molecular mechanisms underlining TRPM4 contribution in EAE, remain largely unclear. In the present study, EAE was induced in WT C57BL/6 N mice using myelin oligodendrocyte glycoprotein 35–55 (MOG_35–55_) and TRPM4 protein and mRNA expression were examined in spinal cord membrane extracts. Our results showed that TRPM4 protein and mRNA are upregulated in EAE, and that their upregulation correlated with disease progression. Moreover, newly-developed TRPM4 inhibitors, named compound 5 and compound 6, were shown to exert a better neuroprotection compared to currently used TRPM4 inhibitors in an in vitro model of glutamate-induced neurodegeneration. These results support the hypothesis that TRPM4 is crucial from early stages of EAE, and suggest that these more potent TRPM4 inhibitors could be used as novel protective therapeutic tools in glutamate-induced neurodegeneration.

**Electronic supplementary material:**

The online version of this article (10.1186/s13041-018-0385-4) contains supplementary material, which is available to authorized users.

## Introduction

Multiple sclerosis (MS) is a multifocal demyelinating disease of the central nervous system (CNS) leading to the progressive destruction of the myelin sheath surrounding axons [1]. The hallmark of demyelinating disease is the formation of the sclerotic plaque, which represents the end of a pathological process involving inflammation, oligodendrocyte depletion, astrocytosis, and neuronal and axon degeneration [2]. Although the mechanisms leading to the development of the disease are not fully understood, numerous evidence indicates that MS is an autoimmune disease, the initiation and progression of which are dependent on an autoimmune response against myelin antigens [3]. However, it is clear that other, non-immunological factors are important in this neurodegenerative process. Recent reports suggested that changes in neuronal ion channel expression and/or function are of pathophysiological importance, although it seems that chronic inflammation is a prerequisite for neurodegeneration at any stage of the disease [4]. One of the central pathophysiological mechanisms leading to axonal and cellular injury is intracellular Na^+^/Ca^2+^ overload to which neurons and oligodendrocytes demonstrate a selective vulnerability [5]. Several ion transport mechanisms that may contribute to toxic Na^+^/Ca^2+^ loading include sodium channels, such as Na_v_1.2 and Na_v_1.6 [6, 7], acid-sensing ion channel 1 (ASIC1) [8, 9] and Na^+^/Ca^2+^ exchanger (NCX) [10, 11]. Among them, recent findings underlined the transient receptor potential melastatin 4 cation channel (TRPM4) as a key player in neuronal degeneration in MS and experimental autoimmune encephalomyelitis (EAE) [12]. TRPM4 is a calcium-activated non-selective cation channel widely expressed in several tissues and reported to be involved in a variety of physiological and pathological processes, including modulation of immune cells activity, such as T-cells [13], mast cells [14] and dendritic cells [15], insulin secretion by pancreatic β cells [16], mechano-transduction in cerebral arteries [17], Ca^2+^ signaling in cancer [18], and several cardiac conduction disorders [19–22] In addition, TRPM4 has been linked to several neurological disorders such as experimental autoimmune encephalomyelitis and multiple sclerosis [12], spinal cord injuries [23], and traumatic brain injuries [24]. Moreover, it has been shown that pharmacological inhibition of TRPM4 using the antidiabetic drug glibenclamide resulted in reduced axonal and neuronal degeneration and attenuated clinical disease scores in EAE [12, 24, 25].

In the present study, we first investigate TRPM4 expression in mouse spinal cords during myelin oligodendrocyte glycoprotein (MOG)_35–55_ peptide-induced EAE. The results show that TRPM4 protein, as well as *Trpm4* gene, are upregulated in EAE, and that this increase correlates with disease progression. Second, newly-developed TRPM4 inhibitors, anthranilic amides named compound 5 and compound 6, are shown to be able to protect neuronal cells from glutamate-induced neurodegeneration, confirming TRPM4 as a potential therapeutic target for glutamate-induced neuronal cell death in EAE.

## Material and methods

### EAE induction

Female 10–12 weeks old WT C57BL/6 N mice were purchased from Harlan Laboratories (Itingen, Switzerland) and immunized subcutaneously in the lower back area with 200 μg MOG_35–55_ (Genscript, Piscataway, NJ, USA) in complete Freund’s adjuvant containing 4 mg/ml heat-killed *Mycobacterium tuberculosis* H37Ra (Difco, FranklinLakes, NJ, USA). 200 ng of pertussis toxin (List Biological Laboratories, Campbell, CA, USA) were administered intra-peritoneally on the day of immunization and 48 h later. The mice were weighted and scored daily as follows: 0 = no clinical deficits; 0.5 = tail paralysis; 1 = hind limb paresis; 1.5 = partial hind limb paralysis; 2 = full hind limb paralysis; 2.5 = full hind limb paralysis and forelimb paresis; 3 = premorbid or dead. Mice with score ≥ 2.5 were sacrificed. As a control, mice were immunized with complete Freund’s adjuvant (CFA) without MOG peptide. At a specific day post immunization, mice were sacrificed under terminal isofluorane anesthesia, transcardially perfused with 100 mL 1X PBS and spinal cords were removed. All animal experiments were approved by the local ethics committee (Amt für Landwirtschaft und Natur des Kantons Bern; BE 139/14).

### Cell transfection and antibody validation

**Fig. 1:**
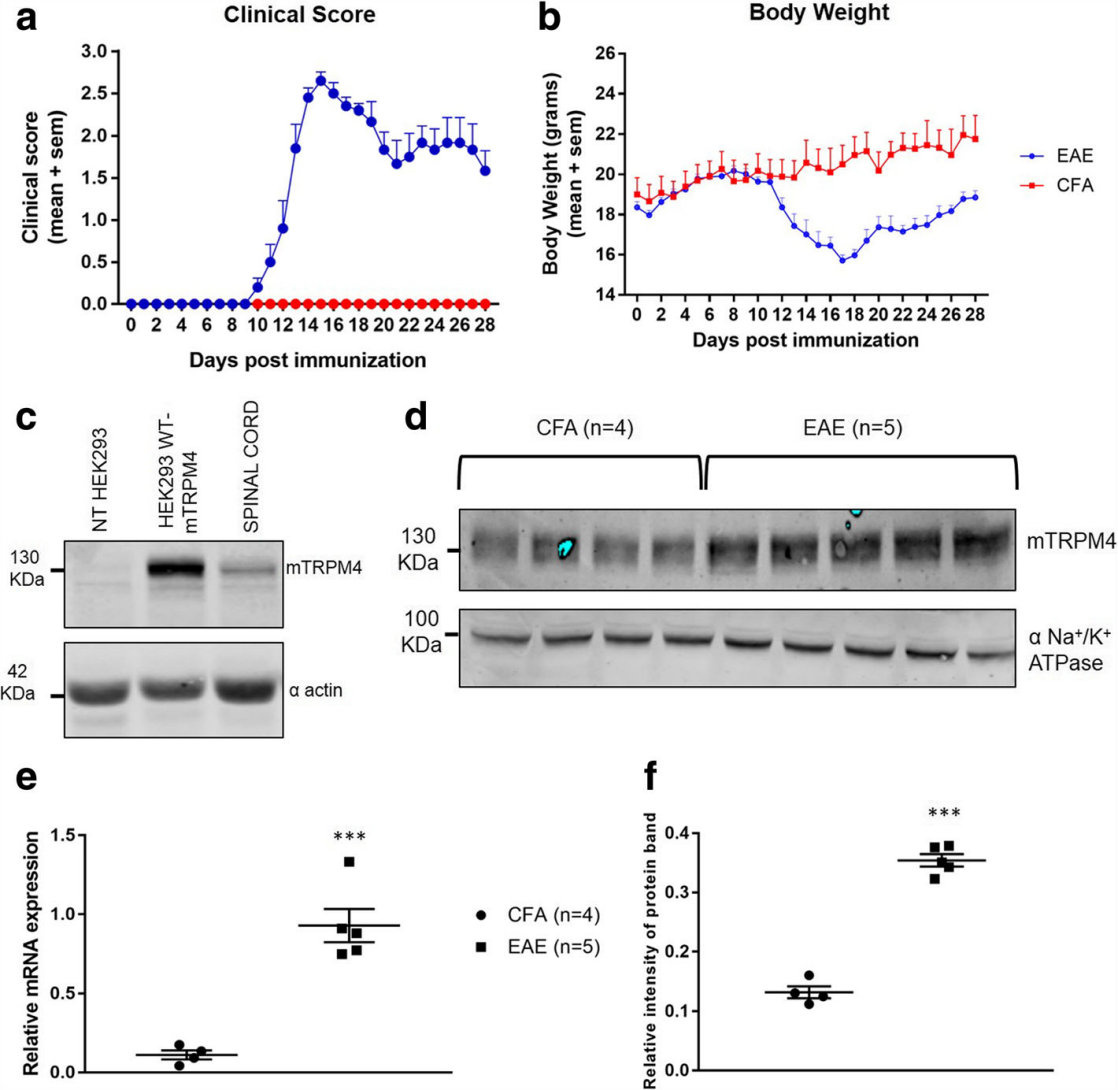
TRPM4 expression analysis in spinal cords from EAE mice. Female C57BL/6 N WT mice were immunized with MOG_35–55_ peptide or with CFA only. Clinical score (**a**) and body weight (**b**) were measured for 28 days after immunization. At day 28 post immunization, spinal cords were extracted and membrane proteins were isolated. **c** Samples from HEK 293 cells transiently transfected with mouse TRPM4 WT plasmid or empty vector (pcDNA4TO) and a spinal cord sample from a healthy WT C57BL/6 N were analyzed for TRPM4 expression, using alpha actin as loading control. A TRPM4 band can be detected at 134 kDa. **d** TRPM4 membrane expression was analyzed with Western Blot, and alpha subunit of Na^+^/K^+^ ATPase is used as a loading control and for TRPM4 normalization (**e**) qPCR analysis on *Trpm4* gene expression in spinal cords from EAE mice and healthy controls. Data are represented as relative mRNA expression and the expression of *Gapdh* is used as reference. **f** Data have been analyzed using unpaired Student’s *t*-test and are represented as mean ± s.e.m. (*** *P* < 0.001)

In order to validate our anti-mouse TRPM4 antibody, human embryonic kidney (HEK293) cells were purchased from Sigma-Aldrich (Darmstadt, Germany), cultured with Dulbecco’s modified Eagle’s culture medium supplemented with 4 mM Glutamine, 10% FBS and a cocktail of streptomycin-penicillin antibiotics and transiently transfected with 300 ng of mouse TRPM4 WT plasmid or empty vector (pcDNA4TO) in a P100 dish (BD Falcon, Durham, North Carolina, USA) mixed with 30 μL of JetPEI (Polyplus transfection, Illkirch, France) and 250 μL of 150 mM NaCl. A spinal cord sample from a healthy C57BL/6 N WT mouse and the transfected cells were then lysed with 1X lysis buffer [50 mM HEPES pH 7.4; 150 mM NaCl; 1.5 mM MgCl_2_; 1 mM EGTA pH 8.0; 10% Glycerol; 1% Triton X-100; 1X Complete Protease Inhibitor Cocktail (Roche, Mannheim, Germany)] and protein expression was investigated using Western Blot technique. The anti- mouse TRPM4 antibody was generated by Pineda Antikorpen (Berlin, Germany) using the following peptide sequence: NH2-VGPEKEQSWIPKIFRKKVC-CONH2. A fraction of the antisera, which was subsequently used in this study, was affinity purified. The results of the antibody validation are shown in Fig. 1c.

### Membrane protein isolation and western blot

For membrane protein expression studies, spinal cords were lysed with 1X lysis buffer and diluted 1 to 5 in a 1 M saccharose solution (1 M HEPES pH 7.4; 1 M saccharose; 1X Complete Protease Inhibitor Cocktail). Tissue lysates were incubated overnight at 4 °C, centrifuged at 3′000 g for 15 min to remove cellular fragments and then ultra-centrifuged at 200′000 g for 40 min. Supernatants were collected and protein concentration was assessed with the Bradford Assay using bovine serum albumin as standard. Eighty μg of protein was loaded on 9% polyacrylamide gels, transferred with the TurboBlot dry blot system (Biorad, Hercules, CA, USA) and detected with anti-mouse TRPM4 (generated by Pineda, Berlin, Germany), anti Na^+^/K^+^ ATPase α1 ab7671 (Abcam, Cambridge, UK) and anti α-actin A2066 (Sigma-Aldrich, Darmstadt, Germany) antibodies using SNAP i.d. (Millipore, Billerica, MA, USA). All the Western Blots have been quantified using Image Studio Lite software from LI-COR Biosciences (Lincoln, NE, USA) and TRPM4 expression was normalized using Na^+^/K^+^ ATPase α1 for membrane fractions and α-actin for total fractions.

### Reverse transcription and quantitative real time PCR

Total RNA isolation was performed using TRIzol Reagent (Applied Biosystems, Foster City, CA, USA) as described by the manufacturer. Concentration and purity of total RNA was determined by optical density measurement using a NanoDrop 2000 spectrophotometer (Thermo Scientific, Waltham, MA, USA). cDNA was synthesized using High Capacity cDNA Reverse Transcription kit (Applied Biosystems, Foster City, CA, USA) and quantitative expression analysis was performed with 7500 Fast Real-Time PCR System (Applied Biosystems, Foster City, CA, USA). Quantification of mRNA expression levels was investigated with TaqMan gene expression assay Mm01205532_m1 for mouse *Trpm4*, using *Gapdh* TaqMan gene expression assay Mm99999915_g1 as a control. Relative expression of the studied gene was calculated with the 2^-ΔΔCt^ method.

### Cell surface biotinylation assay

To study the expression at the plasma membrane, biotinylation assay of membrane proteins was used. In this assay, membrane proteins are labeled with biotin and subsequently immunoprecipitated with streptavidin beads to isolate the protein membrane fraction. Mouse hippocampal HT22 cells (Merck Millipore, Burlington, MA, USA), previously incubated with 1 mM glutamate (Sigma Aldrich, Darmstadt, Germany), were treated with EZlinkTM Sulfo-NHS-SS-Biotin (Thermo Scientific, Waltham, MA, USA) 0.5 mg/mL in cold 1X PBS for 15 min at 4 °C. Subsequently, the cells were washed twice with 200 mM Glycine in cold 1X PBS and twice with cold 1X PBS to inactivate and remove the excess of biotin, respectively. The cells were then lysed and centrifuged at 16′000 g at 4 °C for 15 min. Two milligrams of the supernatant were incubated with 50 μL Streptavidin Sepharose High Performance beads (GE Healthcare, Uppsala, Sweden) for 2 h at 4 °C, while forty μg of protein were kept for the input fraction. The beads were subsequently washed five times with 1X lysis buffer before elution with 50 μL of 2X NuPAGE sample buffer (Invitrogen, Carlsbad, CA, USA) plus 100 mM DTT at 37 °C for 30 min.

### Cellular neuroprotection assays

To assess the neuroprotective effect of TRPM4 blockers, HT22 cells were incubated with different TRPM4 inhibitors or 1% of their dissolving medium DMSO as control for 2 h before incubation with 5 mM glutamate. Two well known TRPM4 blockers, 9-phenantrol and glibenclamide, as well as newly-developed blockers, such as 4-Chloro-2-(2-(2-chlorophenoxy)acetamido) benzoic acid, also called compound 5 or CBA, and 4-Chloro-2-(2-(naphthalene-1-yloxy) acetamido) benzoic acid, also called compound 6 or NBA, were used at 5 and 10 μM concentrations. Cell viability was assessed using Tripan blue dye and the number of dead cells was measured using Countess II FL Automated Cell Counter (Thermo Scientific, Waltham, MA, USA). Values were expressed as the percentage of survival compared to untreated controls. Using the same procedure, cell integrity was assessed using LDH-Cytotoxicity Assay kit II (Abcam, Cambridge, UK) according to manufacturer instructions and values were expressed as percentage of controls.

### Statistical analysis

Western blot, qPCR and cell viability experiments were analyzed using a Student’s *t-*test or one-way ANOVA with Sidak’s correction for multiple comparison, with a *p*-value < 0.05 considered as significant. Data are represented as the mean ± s.e.m. Correlation analyses were performed with Spearman’s correlation analysis test. GraphPad Prism version 7 (GraphPad Software, San Diego, CA, USA) was used for the analysis. Statistical post-hoc power calculation has been performed using Stata 14.2 in experiments using three mice, and the results are uploaded as Additional file 1.

## Results

### Membrane expression of TRPM4 is increased in EAE spinal cords

Female C57BL/6 N mice were immunized with MOG_35–55_ peptide or with CFA only as described in the ‘Material and Methods’ section. Mice immunized with the MOG_35–55_ peptide exhibit the first clinical symptoms and body weight loss around 10 days post immunization and a peak of the disease was reached at day 15, while CFA-immunized animals did not show any relevant clinical phenotype (Fig. 1a and b). At day 28 post immunization, animals were sacrificed and the spinal cords were removed for mRNA and protein expression analysis. Membrane expression of TRPM4 was assessed, resulting in a significant higher protein band intensity in EAE animals compared to CFA controls (Fig. 1d and f). We then performed qPCR on the same spinal cord samples to determine whether this increase in protein expression may have been due to increased mRNA levels. Results in Fig. 1e revealed a significant increase in *Trpm4* gene expression in EAE compared to healthy controls.

### TRPM4 expression in spinal cords in EAE correlates with disease stages

**Fig. 2:**
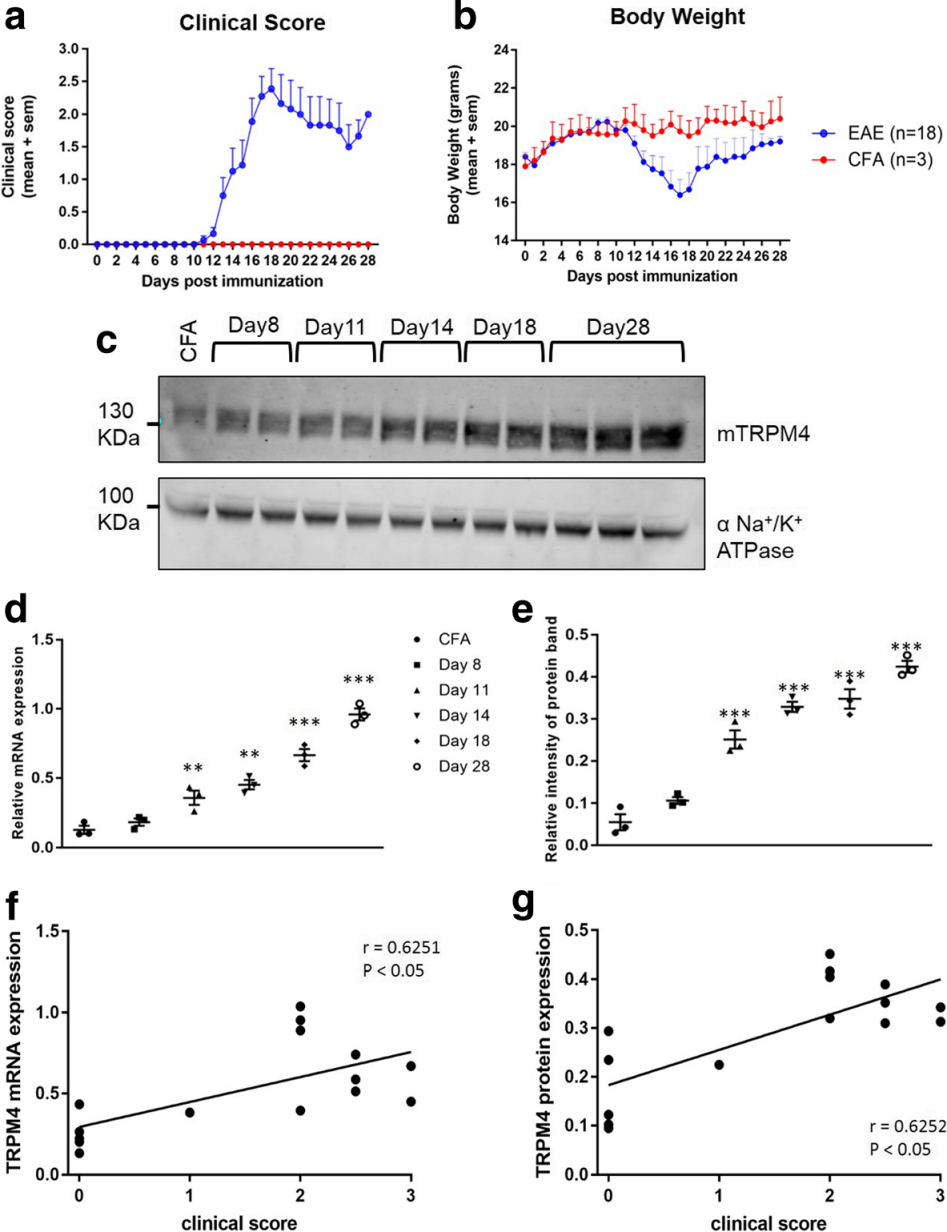
TRPM4 expression in spinal cords from EAE mice correlates with disease stages. Female C57BL/6 N WT mice were immunized with MOG_35–55_ peptide or with CFA only and sacrificed at different days post immunization reflecting different EAE stages. Clinical score and body weight are represented in panel **a** and **b** respectively. **c** and **e** TRPM4 protein expression at different EAE stages is shown as a relative intensity of protein band and alpha subunit of Na^+^/K^+^ ATPase is used as a loading control and for TRPM4 normalization. **d** qPCR analysis on *Trpm4* gene expression in spinal cords from EAE mice and healthy control at different EAE stages. Data are represented as relative mRNA expression and the expression of *Gapdh* is used as reference. Data have been analyzed using one-way ANOVA with Sidak’s correction for multiple comparison test and are represented as mean ± s.e.m. (** *P* < 0.01; *** *P* < 0.001). Correlation between clinical score and TRPM4 mRNA (**f**) or TRPM4 protein expression (**g**) is calculated using Spearman’s correlation analysis. Spearman correlation coefficients (r) and their corresponding *p* values are shown

To further investigate the involvement of TRPM4 in EAE, 18 female C57BL/6 N mice were immunized as described previously and sacrificed on different days post immunization reflecting different stages of the disease, as shown in Table 1. EAE clinical score and body weight are shown in Fig. 2a and b. After sacrifice, spinal cords were collected and membrane protein lysates as well as mRNA were prepared. Interestingly, an increase of TRPM4 expression, compared to CFA controls, was already detected at pre-clinical stage of EAE, and it became more significant at the EAE acute phase. Consistently, TRPM4 protein expression continued to increased and reached the highest value at the chronic stage of EAE (Fig. 2c and e). Together, TRPM4 mRNA expression was analyzed in the different EAE phases and also resulted to correlate with disease stage (more advanced EAE progression = higher *Trpm4* gene expression), as reported in Fig. 2d. Correlation analysis between disease stage and TRPM4 mRNA expression, as well as protein expression, has been performed, and it is shown in Fig. 2f and g, respectively. Spearman’s analysis resulted in a coefficient (r) of approximately 0.6, supporting a correlation between disease progression and TRPM4 mRNA and protein expression.

**Table 1:** Mice sacrifice at different EAE stages

	Day of sacrifice	Average clinical score	EAE stage
Mice 1–3	Day 8	0	Immune activation but no EAE symptoms
Mice 4–6	Day 11	0.5	Weight loss and very early EAE symptoms
Mice 7–10	Day 14	1.5	Confirmed EAE symptoms
Mice 11–14	Day 18	2.5	EAE peak
Mice 15–18	Day 28	2.0	Chronic EAE symptoms

### Imbalanced glutamate metabolism does not alter TRPM4 expression

In a recent work, a mechanism of neurodegeneration involving TRPM4 has been proposed and resulted to be related to imbalanced glutamate metabolism. In inflammatory conditions (such as in multiple sclerosis), astrocytes release high amounts of glutamate, which in turn induces neurodegeneration by eliciting Ca^2+^ and Na^+^ influx. The increase in intracellular Ca^2+^ activates TRPM4, and the TRPM4-mediated inward current causes cell swelling and cell death [12]. In addition, it was reported that hippocampal neurons from TRPM4 KO mice were unaffected by glutamate exposure [12], consistent with a model that neuronal death in EAE is mainly driven by glutamate.

HT22 mouse hippocampal cells have been reported to be a good model for glutamate-induced neurodegeneration with involvement of TRPM4 [26]. In this work by Cho and colleagues, HT22 cells exhibited an endogenous TRPM4 activation after incubation with glutamate, and TRPM4 currents resulted to be inhibited by 9-phenantrol [26].

**Fig. 3:**
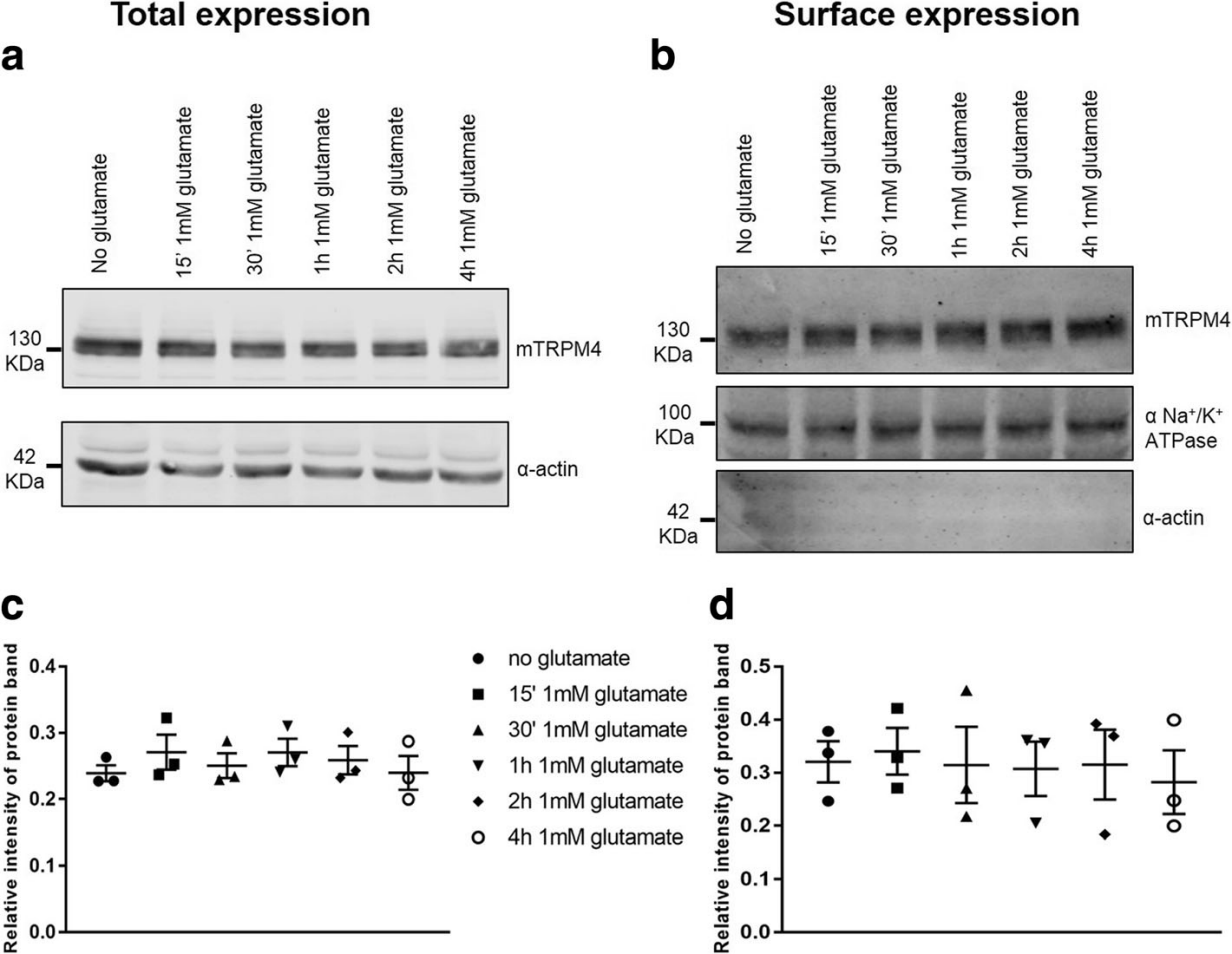
Incubation of HT22 cells with 1 mM glutamate does not alter TRPM4 expression. HT22 cells were incubated with 1 mM glutamate and harvested at different time points after glutamate addition. TRPM4 total (**a**) as well as surface (**b**) expression were evaluated, using alpha actin and alpha subunit of Na^+^/K^+^ ATPase as loading controls and for TRPM4 normalization. **c** and **d** Total and surface TRPM4 protein expression are shown as a relative intensity of protein band. No significant difference is reported (data are of three independent experiments)

To investigate whether TRPM4 expression was modulated by imbalanced glutamate metabolism, we incubated HT22 cells with 1 mM glutamate and harvested them at different time points after glutamate addition. As shown in Fig. 3, addition of glutamate did not affect TRPM4 protein expression, both in the total fraction (Fig. 3a and c) and at the cell surface (Fig. 3b and d). Hence, this suggested that glutamate is involved in increased TRPM4 activation, but does not have any role in increased TRPM4 expression at the cell membrane.

### TRPM4 specific inhibitors attenuate in vitro glutamate-induced neurodegeneration

Recently, due to a lack in specific TRPM4 inhibitors, more selective and potent compounds have been synthesized and screened using a fluorescence cell-based screening assay [27]. From this screening, two promising anthranilic amides analogs of flufenamic acid, named compound 5 (4-Chloro-2-(2-(2-chlorophenoxy)acetamido) benzoic acid, CBA) and compound 6 (4-Chloro-2-(2-(naphthalene-1-yloxy) acetamido) benzoic acid, NBA), have been identified, and resulted to have an IC_50_ of 1.5 ± 0.1 μM and 0.4 ± 0.3 μM respectively [27].

**Fig. 4:**
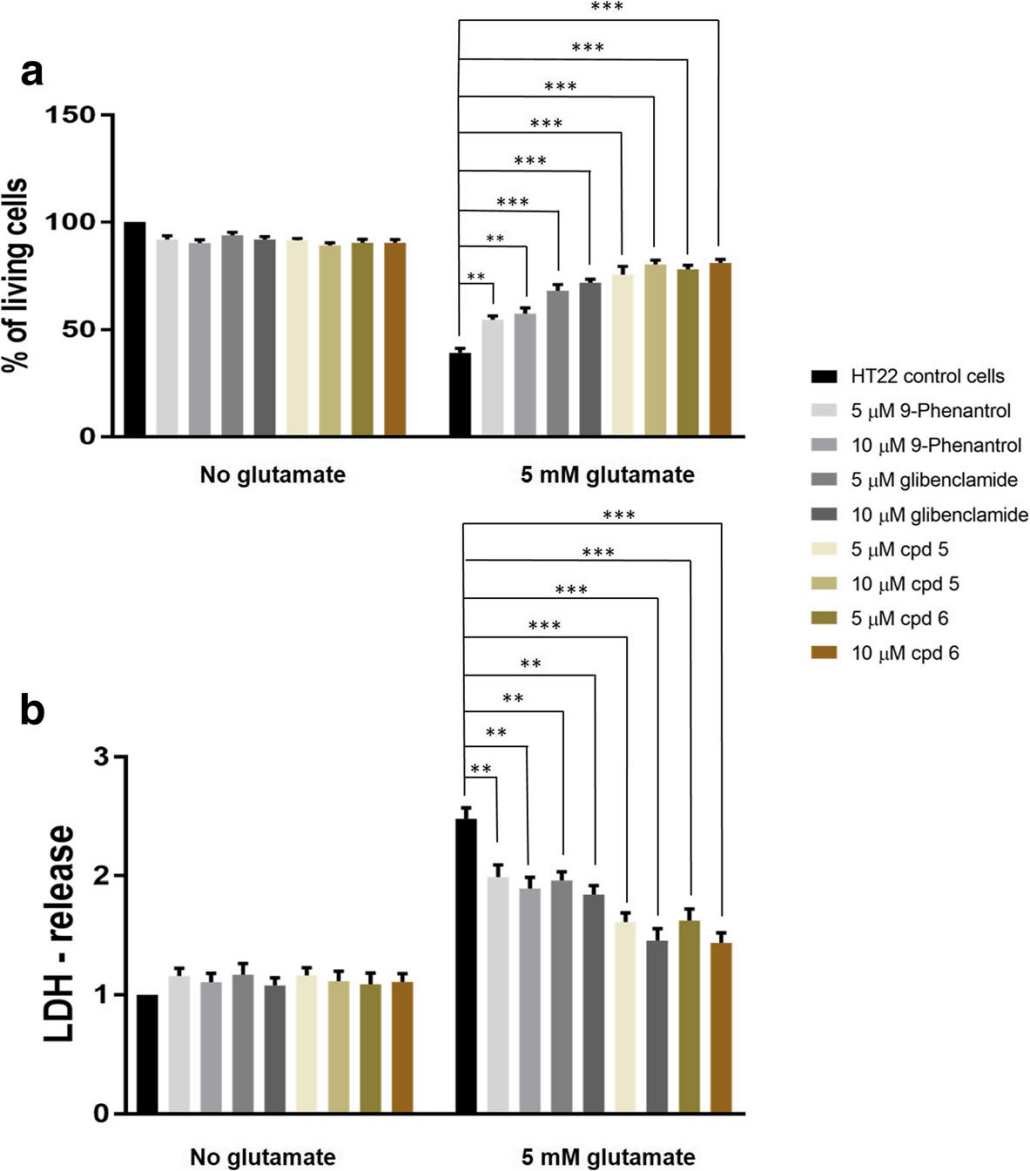
Newly-developed TRPM4 inhibitors better attenuate glutamate-induced neurodegeneration compared to commonly used compounds. HT22 cells were incubated with 5 mM glutamate after pre-incubation with 5 μM and 10 μM of commonly used TRPM4 inhibitors as well as newly-developed ones. The bar graphs showed the results of cell viability (**a**) and LDH-release assays (**b**), where glutamate-induced cell death and membrane disruption were prevented dose-dependently, with newly-developed compounds exerting a more beneficial effect. Data have been analyzed using one-way ANOVA with Sidak’s correction for multiple comparison test and are represented as mean ± s.e.m. (** *P* < 0.01; *** *P* < 0.001; *n* = 10 for each condition)

We then tested whether the newly-developed TRPM4 inhibitors could better attenuate glutamate-induced cell death compared to commonly used compounds, such as 9-phenantrol and glibenclamide, both shown to exert a neuroprotective effect by blocking TRPM4 activity [12, 25, 26, 28]. Neuronal cell death was induced in vitro by incubating HT22 cells with 5 mM glutamate after a 2 hours pre-incubation with 5 or 10 μM 9-phenantrol, glibenclamide, and the two newly-developed compound 5 and compound 6. All compounds resulted to be well tolerate and did not induce any cell death or loss of integrity in HT22 cells without glutamate (Fig. 4a and b). As shown in Fig. 4, all TRPM4 inhibitors protected neuronal cells against cell death and loss of integrity induced by 5 mM glutamate in a dose dependent manner. Moreover, compound 5 and compound 6 exerted a more beneficial effect compared to 9-phenantrol or glibenclamide, with a reduction of dead cells and LDH release of ~ 40% (Fig. 4a and b).

## Discussion

Multiple sclerosis is the most common autoimmune disorder of the central nervous system, affecting approximately 2.5 million people worldwide [1]. It is primarily an inflammatory disorder of the brain and spinal cord in which focal lymphocytic infiltration leads to damage of myelin and axons; however, a growing body of evidence suggests that non-immunological factors, such as changes in neuronal ion channel expression and/or function, are of pathophysiological importance [29]. In a recent study by Schattling and colleagues, TRPM4 was found to be expressed in axons and neurons of mice and humans, and suggested to mediate neurodegeneration by contributing in glutamate-induced cell swelling and cell death without affecting the immune response triggered in the EAE model [12]. In addition, authors showed that hippocampal neurons from TRPM4 KO mice were unaffected by glutamate exposure, suggesting that glutamate is a major contributor to neuronal cell death in EAE involving TRPM4 [12].

In the present study, we reported that TRPM4 is not only overexpressed in spinal cords from EAE mice compared to healthy controls, but that its increased expression correlated with disease progression. We then demonstrated that this increase in TRPM4 membrane expression is due to an increase in *Trpm4* gene expression. Moreover, it has been shown that during the late stages of EAE, there is a transition from an inflammatory phenotype to a non-remitting neurodegenerative profile with CNS atrophy [30]. For this reason, newly-developed TRPM4 inhibitors have been tested in vitro to attenuate glutamate-induced neurodegeneration, resulting in an increased cell integrity and survival, compared to well-known TRPM4 blockers.

It is indeed evident that two parallel mechanisms involving TRPM4 are occurring in EAE: a first mechanism leading to increased TRPM4 expression at the cell membrane and a subsequent one leading to increased channel activation triggered by glutamate. It has been reported that during EAE-dependent neuroinflammation, astrocytes activation causes high glutamate release leading to increased extracellular glutamate concentration. High glutamate levels induce neurodegeneration by eliciting Na^+^ and Ca^2+^ influx which, in turn, activates TRPM4 [31]. However, based on our findings, an increase in extracellular glutamate does not alter TRPM4 membrane expression, but only modulates its function. Together with an increase in TRPM4 protein expression, *Trpm4* gene seems to be substantially upregulated, and its upregulation correlates with the degree of inflammation and neurodegeneration in EAE. Hence, an additional pathway leading to increased *Trpm4* gene expression is likely to occur.

Many gene expression studies have been undertaken to look at the gene transcript levels in MS and EAE. In general, genes showing variable expression included mainly immunological and inflammatory genes, such as genes encoding interleukin-6, interleukin-17 and interferon-gamma [32, 33]. Interestingly, several genes encoding proteins that play critical roles in ion homeostasis, mitochondrial function and impulse conduction have been shown to be dysregulated. In particular, transcript and protein levels of the Ca^2+^ ATPase 2 (PMCA2), an ion pump involved in Ca^2+^ extrusion, were dramatically decreased in coincidence with the onset of clinical symptoms in a rat model of EAE [34]. Together, genes encoding sodium channels have been reported to be dysregulated in inflammatory conditions [34, 35]. The mechanism by which this dysregulation of sodium channels occurred remains elusive. It has been hypothesized that mediators such as nerve growth factor (NGF) or cytokines released at the site of inflammation might be transported to the innervating perikarya, resulting in the stimulation of channel synthesis [36]. This mechanism proposed by Mandel and colleagues could explain the gene upregulation of sodium channels, and could be used to explain *Trpm4* upregulation in EAE. However, further studies are required to address the exact molecular mechanism leading to *Trpm4* gene expression increase in EAE.

Together with an increase in *Trpm4* gene expression, subsequent mechanisms for increased TRPM4 membrane expression and trafficking independent from the transcriptional machinery can occur. It was reported that in cardiac conduction disturbances with involvement of TRPM4, where several genetic variants of the channel were leading to an increase in the membrane protein expression without affecting the gene expression [19, 22]. This was due to altered half-life and/or post translational modification (mainly SUMOylation) affecting the trafficking and/or stability of the protein [19, 22]. In addition, TRPM4 was reported to be overexpressed in prostate cancer, where its increased expression promoted the stabilization and activity of β-catenin enhancing cell proliferation, but the underlying mechanisms have not yet been clarified [18]. Further, it has been shown that a glutamate receptor-mediated intracellular calcium rise activates the protein kinase C (PKC) pathway, which could in turn influence TRPM4 trafficking to the cell membrane [37]. Moreover, an increased sulfonylurea receptor 1 (SUR1)-TRPM4 interaction has been reported in spinal cords from EAE mice, suggesting a possible role of this interaction in increased TRPM4 surface expression [25]. Lastly, a recent work described that 14–3-3γ depletion reduced TRPM4 expression and attenuated glutamate-induced neuronal cell death [26]. Hence, post-translational mechanisms are likely to occur, together with increased *TRPM4* gene expression, to enhance TRPM4 trafficking to the cell membrane. Once at the membrane, TRPM4 is over-activated by increased Ca^2+^ influx triggered by glutamate.

Expression of TRPM4 has been reported in neurons, and they could be the origin of TRPM4 overexpression in spinal cords of EAE mice. Alternatively, an increased TRPM4 expression could be due to differences in CNS-infiltrating immune cells, although Schattling and colleagues did not report any change in infiltrating cells between WT and TRPM4 KO mice after EAE induction [12].

TRPM4 was found to be expressed in astrocytes and to be involved in astrocyte swelling in several pathological conditions, such as brain edema [38], and glaucoma [39]. In EAE and multiple sclerosis, TRPM4 channels were reported to be predominantly expressed by reactive astrocytes in pathologically involved tissues that exhibit a significant inflammatory burden, such as spinal cords [25]. Based on this observation, it was hypothesized that the channel contributed to astrocyte-mediated inflammation in EAE, and that in vivo treatment with TRPM4 blockers would ameliorate clinical symptoms. Hence, astrocytes could possibly be the cell population of the spinal cord that contributes to increased TRPM4 expression.

In conclusion, accumulating evidence indicates a crucial role of TRPM4 in EAE as well as in multiple sclerosis, confirming TRPM4 as a strong potential therapeutic target in these diseases. Despite that, additional experiments are required to better understand that molecular pathways of TRPM4 in neurological disorders, such as multiple sclerosis.

### Limitations of the study

This work is a mostly confirmatory study about the involvement of TRPM4 in EAE, and it shows that TRPM4 inhibition can ameliorate glutamate-induced neurodegeneration; however, one can note some limitations. First, the assumption that glutamate is the main contributor to neuronal cell death in EAE involving TRPM4 remains a hypothesis, since, to date, we have no tools to demonstrate it in vivo and to make a direct link between the in vitro glutamate study and the in vivo EAE study. Second, the WBs are membrane fraction preparations from whole spinal cord tissue. Therefore, we cannot identify the specific cell population responsible for TRPM4 upregulation. Further studies are needed to address these pending questions.

## Additional file

Additional file 1: Post-hoc power calculation. Post-hoc power calculation has been performed using Stata 14.2 to calculate statistical power of experiments with three mice, both for mRNA as well as protein expression studies. Results showed a 90% power with N/group = 2. (PDF 236 kb)

## Abbreviations

ASIC1: acid-sensing ion channel 1
Ca^2+^: calcium
CFA: complete Freund’s adjuvant
CNS: central nervous system
cpd: compound
EAE: experimental autoimmune encephalomyelitis
MOG: myelin oligodendrocyte glycoprotein
MS: multiple sclerosis
Na^+^: sodium
NCX: Na^+^/Ca^2+^ exchanger
NGF: nerve growth factor
PKC: protein kinase C
PMCA2: Ca^2+^ ATPase 2
SUR1: sulfonylurea receptor 1
TRPM4: transient receptor potential melastatin 4
WT: wild-type
