# Supplementary material for: The ion channel TRPM4 in murine experimental autoimmune encephalomyelitis and in a model of glutamate-induced neuronal degeneration
Source: Mol Brain. 2018 Jul 11;11:41. doi: 10.1186/s13041-018-0385-4 (PMC6042389; doi:10.1186/s13041-018-0385-4)
Supplement: Supplementary file 1 — Post-hoc power calculation. Post-hoc power calculation has been performed using Stata 14.2 to calculate statistical power of experiments with three mice, both for mRNA as well as protein expression studies. Results showed a 90% power with N/group = 2. (PDF 236 kb) [file 13041_2018_385_MOESM1_ESM.pdf]

Statistical Power Calculation of WB experiment (Figure 2C and E)

```
. power twomeans 11 40, sd1(3) sd2(2) alpha(.05) power(0.9)

Performing iteration ...

Estimated sample sizes for a two-sample means test
Satterthwaite's t test assuming unequal variances
Ho: m2 = m1  versus  Ha: m2 != m1

Study parameters:

        alpha =    0.0500
        power =    0.9000
        delta =   29.0000
           m1 =   11.0000
           m2 =   40.0000
          sd1 =    3.0000
          sd2 =    2.0000

Estimated sample sizes:

            N =         4
  N per group =         2
```

Statistical Power Calculation of qPCR experiment (Figure 2D)

```
. power twomeans 36 96, sd1(7) sd2(6) alpha(.05) power(0.9)

Performing iteration ...

Estimated sample sizes for a two-sample means test
Satterthwaite's t test assuming unequal variances
Ho: m2 = m1  versus  Ha: m2 != m1

Study parameters:

        alpha =    0.0500
        power =    0.9000
        delta =   60.0000
           m1 =   36.0000
           m2 =   96.0000
          sd1 =    7.0000
          sd2 =    6.0000

Estimated sample sizes:

            N =         4
  N per group =         2

.
```

We artificially reduced the difference and transformed the values multiplying by 100, because the estimated power was not achievable given the domain limits of the power function.
